# Pretreatment with adalimumab reduces ventilator-induced lung injury in an experimental model

**DOI:** 10.5935/0103-507X.20200010

**Published:** 2020

**Authors:** Enrique Correger, Josefina Marcos, Graciela Laguens, Pablo Stringa, Pablo Cardinal-Fernández, Lluis Blanch

**Affiliations:** 1 Workgroup in Experimental Pulmonary Pathophysiology, Faculty of Medicine, National University of La Plata - La Plata, Argentina.; 2 Critical Care Department, Hospital El Cruce - Buenos Aires, Argentina.; 3 Rheumatology Department, IPENSA La Plata - La Plata, Argentina.; 4 Pathology A Chair, Faculty of Medicine, National University of La Plata - La Plata, Argentina.; 5 Organ Transplant Laboratory, School of Medicine, National University of La Plata - La Plata, Argentina.; 6 Emergency Department, Hospital Universitario HM Sanchinarro - Madrid, Spain.; 7 Critical Care Center, Corporació Sanitària Parc Taulí - Sabadell, Barcelona, Spain.

**Keywords:** Adalimumab/administration & dosage, Respiration, artificial, Respiratory failure, Ventilator-induced lung injury, Adalimumabe/administração & dosagem, Respiração artificial, Insuficiência respiratória, Lesão pulmonar induzida por ventilação mecânica

## Abstract

**Objective:**

To determine whether adalimumab administration before mechanical ventilation reduces ventilator-induced lung injury (VILI).

**Methods:**

Eighteen rats randomized into 3 groups underwent mechanical ventilation for 3 hours with a fraction of inspired oxygen = 0.40% including a low tidal volume group (n = 6), where tidal volume = 8mL/kg and positive end-expiratory pressure = 5cmH2O; a high tidal volume group (n = 6), where tidal volume = 35mL/kg and positive end-expiratory pressure = 0; and a pretreated + high tidal volume group (n = 6) where adalimumab (100ug/kg) was administered intraperitoneally 24 hours before mechanical ventilation + tidal volume = 35mL/kg and positive end-expiratory pressure = 0. ANOVA was used to compare histological damage (ATS 2010 Lung Injury Scoring System), pulmonary edema, lung compliance, arterial partial pressure of oxygen, and mean arterial pressure among the groups.

**Results:**

After 3 hours of ventilation, the mean histological lung injury score was higher in the high tidal volume group than in the low tidal volume group (0.030 *versus* 0.0051, respectively, p = 0.003). The high tidal volume group showed diminished lung compliance at 3 hours (p = 0.04) and hypoxemia (p = 0,018 versus control). Pretreated HVt group had an improved histological score, mainly due to a significant reduction in leukocyte infiltration (p = 0.003).

**Conclusion:**

Histological examination after 3 hours of injurious ventilation revealed ventilator-induced lung injury in the absence of measurable changes in lung mechanics or oxygenation; administering adalimumab before mechanical ventilation reduced lung edema and histological damage.

## Abstract (pt)

**Objetivo:**

Determinar se a administração de adalimumabe previamente à ventilação mecânica reduz a lesão pulmonar induzida por ventilação mecânica.

**Métodos:**

Randomizaram-se 18 ratos em três grupos submetidos à ventilação mecânica por 3 horas com uma fração inspirada de oxigênio de 0,40%. Os três grupos foram assim caracterizados: um grupo com baixo volume corrente (n = 6), no qual se utilizaram volume corrente de 8mL/kg e pressão expiratória final positiva de 5cmH_2_O; um grupo com alto volume corrente (n = 6), no qual se utilizaram volume corrente de 35mL/kg e pressão expiratória final positiva de zero; e um grupo pré-tratado com alto volume corrente (n = 6), no qual se administraram adalimumabe (100µg/kg) por via intraperitoneal 24 horas antes do início da ventilação mecânica, volume corrente de 35mL/kg e pressão expiratória final positiva de zero. Realizou-se ANOVA para comparação de dano histológico (com utilização de escores segundo o *ATS 2010 Lung Injury Scoring System*), edema pulmonar, complacência pulmonar, pressão parcial de oxigênio arterial e pressão arterial média entre os grupos.

**Resultados:**

Após 3 horas de ventilação, o escore médio de lesão histológica pulmonar foi mais elevado no grupo com alto volume corrente do que no grupo com baixo volume corrente (0,030 versus 0,0051; p = 0,003). O grupo com alto volume corrente demonstrou complacência pulmonar diminuída após 3 horas (p = 0,04) e hipoxemia (p = 0,018 versus controle). O grupo alto volume corrente tratado previamente teve melhora do escore histológico, principalmente devido à redução significante da infiltração leucocitária (p = 0,003).

**Conclusão:**

O exame histológico após 3 horas de ventilação lesiva revelou lesão pulmonar induzida por ventilação mecânica na ausência de modificações mensuráveis na mecânica pulmonar e na oxigenação; a administração de adalimumabe antes da ventilação mecânica diminuiu o edema pulmonar e o dano histológico.

## INTRODUCTION

Acute respiratory distress syndrome (ARDS) is among the main indications for mechanical ventilation (MV); ARDS and MV a significant impact on public health, with mortality rates between 35% and 65%.^(1,2)^ Although lifesaving, MV can also cause lung injury or aggravate pre-existing injury. This ventilator-induced lung injury (VILI), characterized by pulmonary edema and inflammation, is indistinguishable from acute lung injury (ALI) of other etiologies.^(3)^ Biophysical forces are responsible for the abnormal lung physiology, causing a proinflammatory state.^(4)^ The inflammatory response results in the release of cytokines, leukocyte recruitment to the lung parenchyma, and a marked increase in vascular permeability, which trigger pulmonary edema and surfactant dysfunction, leading to decreased respiratory system compliance (CL) and impaired gas exchange.^(5-8)^

Lung-protective ventilation strategies based on using a low tidal volume (Vt) can reduce but not eliminate the risk of VILI.^(9-11)^ These strategies are associated with decreased mortality in patients with ARDS.^(12)^ Other strategies to attenuate lung inflammation may also help reduce VILI. The proinflammatory cytokine tumor necrosis factor alpha (TNF-α) has been consistently implicated in the pathogenesis of VILI in both experimental and clinical studies.^(13-17)^ Moreover, neutrophil recruitment is substantially attenuated in TNF-α receptor-knockout mice and in mice treated with an intratracheally administered anti-TNF-α antibody.^(16)^

Adalimumab is a fully human monoclonal antibody (anti-TNF IgG1) that specifically binds to soluble and transmembrane TNF-α with high affinity, neutralizing the biological function of TNF-α by blocking its interaction with the cell surface receptors p55 and p75 TNF-α. Binding of antibodies with transmembrane TNF-α induces an intracellular signaling cascade that results in the release of intracellular calcium, the production of cytokines, and the expression of E-selectin, inducing apoptosis.^(18-21)^ Animal studies have shown that inducing apoptotic pathways limits lung inflammation.^(6,7)^

We aimed to determine the effects of blocking TNF-α by pretreatment with adalimumab before MV on clinical and histological parameters related to pulmonary edema.

## METHODS

### Animals and monitoring

We studied 18 adult male Wistar rats (mean weight: 340 ± 15g) housed under controlled macro- and microenvironmental conditions with access to food and water *ad libitum* before the experiment. Rats were anesthetized with a combination of ketamine (80mg/Kg) and xylazine (20mg/kg) administered intraperitoneally. Then, lidocaine (10mg/kg) was applied as a local anesthetic in the subcutaneous tissue of the ventral neck region. After the appropriate depth of anesthesia was established, the animals were placed in a supine position on a heating pad to avoid hypothermia during the procedure. After an incision was made in the ventral neck region from the caudal jaw to the first third of the thorax, the left carotid artery and the right jugular vein were dissected and cannulated with 24G Teflon catheters. The arterial line was connected to the multiparameter monitor Dyne MCO-300-07 (Argentina) to measure mean arterial pressure (MAP) during the procedure. To maintain adequate hydration, the venous line was connected to an infusion pump administering a normal saline solution (10mL/h) throughout the study.

Rats were tracheotomized, and a cannula was introduced into the tracheostomy and connected to a mechanical ventilator (Neumovent GraphNet neo TECME^®^).

### Experimental protocol

The university’s Animal Research Ethics Committee approved the experimental protocol. After initial monitoring, all animals were ventilated for 20 minutes with the following settings: volume control ventilation, adjusting Vt to 8mL/kg, positive end-expiratory pressure (PEEP) of 5cmH_2_O, square wave, respiratory rate of 80 breaths per minute and fraction of inspired oxygen (FiO_2_) of 0.40 without an inspiratory pause. The inspiratory time of 0.27 seconds and respiratory rate were adjusted afterwards to maintain the partial pressure of arterial carbon *dioxide (*PaCO_2_) between 45 - 55mmHg. Then, rats were randomized to one of three ventilation strategies: (1) the low Vt (LVt) group (n = 6): Vt of 8mL/kg and 5cmH_2_O of PEEP, (2) High Vt (HVt) group (n = 6): Vt of 35mL/kg and zero PEEP, or (3) pretreated HVt group (n = 6): adalimumab (100µg/kg, 50mg/mL in sterile water, diluted in a physiological solution to a concentration of 500µg/mL^(21)^) administered by intraperitoneal injection 24 hours before starting MV with a Vt of 35mL/kg and zero PEEP. The animals in the LVt and HVt groups were administered equivalent amounts of saline 24 hours before starting MV. After randomization, all animals were ventilated for 3 hours at a respiratory rate of 80 breaths per minute or with adjusting the respiratory frequency to maintain intended PaCO_2_ values, an FIO_2_ of 0.40 without an inspiratory pause, and the Vt and PEEP according to the group. When the Vt was increased for the animals in the HVt and pretreated HVt groups, dead space ventilation was increased to obtain comparable values ​​of PaCO_2_ among the three groups. At the end of MV, the rats were euthanized by exsanguination. Immediately after death, the lungs were removed; the left lung was instilled with 10% formaldehyde for histopathological study, and the right lower lobe was removed and weighed. The right lower lobe was placed in an oven at 60°C for 48 hours and weighed again; the difference between the wet weight and dry weight (W/D ratio) was calculated.

### Measurements and calculations

To measure PEEP, plateau pressure (Ppl), and Vt for the calculation of lung compliance [CL = Vt/Ppl - PEEP], we used neonatal lung transducers (Pulmonary Monitor CP-100, Bicore Monitoring Systems; Irvine, CA, USA). At 0 and 180 minutes, we measured MAP, Ppl, and Vt, and we used a portable blood analyzer (i-Stat Handheld, Abbot Inc.; Princeton, NJ, USA) to measure the partial pressure of arterial *oxygen (*PaO_2_), PCO_2_, and pH in blood from the carotid artery catheter.

### Histological analysis

Three sections of each animal’s lungs from above and below the hilum were selected for histological analysis and stained with hematoxylin and eosin. A pathologist, who was blinded to treatment received, used the ATS 2010 Lung Injury Scoring System to assess the following parameters: (A) neutrophils in the alveolar space, (B) neutrophils in the interstitial space, (C) hyaline membrane formation, (D) intraalveolar proteinaceous material, and (E) alveolar septal thickening; score = [(20 × A) + (14 × B) + (7 × C) + (7 × D) + (2 × E)]/(number of 100× fields).^(7)^

### Apoptosis

Two investigators used light microscopes at 40× magnification to evaluate two hematoxylin and eosin-stained slides from each animal’s lungs to evaluate morphological criteria of apoptosis, defined as dense condensation of the nuclei of polymorphonuclear leukocytes or the presence of polymorphonuclear leukocytes inside macrophages. These findings are reported as the percentage of apoptotic cells.

### Statistical analysis

Data are expressed as the mean ± standard deviation (SD). To compare variables among groups, we used ANOVA or Student’s t test as appropriate, considering p < 0.05 significant. Statistix 9 (Analytical Software; Tallahassee, FL, USA) was used for all analyses.

Graphics were performed using GraphPad software version 5.0 (San Diego, CA).

## RESULTS

**Figure 1:**
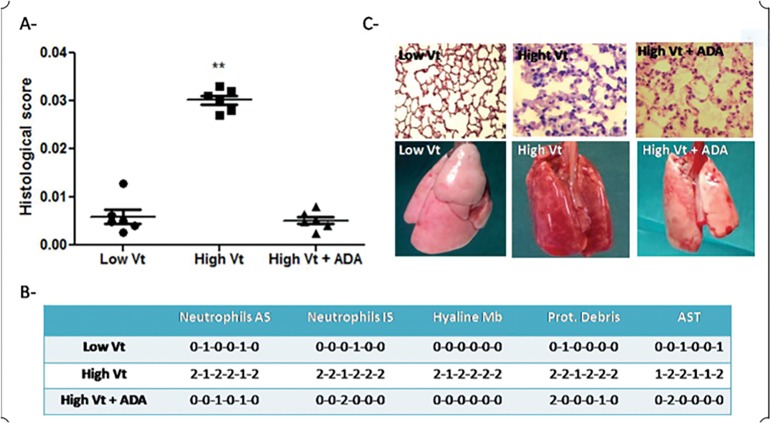
Macro and microscopic evaluation of lungs. (A) Histological scores of the three groups: low tidal volume, high tidal volume, and adalimumab + high tidal volume. (B) Discrimination of the American Thoracic Society Lung Injury Scoring System variables. (C) Top images: light microscopy, hematoxylin and eosin, original magnification: 40x. Left (low tidal volume group): normal lung architecture tissue. Middle (high tidal volume group): lungs with intraalveolar and interstitial leukocyte infiltration, hyaline membrane formation and septal thickening. Right (adalimumab + high tidal volume group): septal thickening with reduced leukocyte infiltration. Bottom pictures: Gross examination. Left (low tidal volume): normal lungs. Middle (high tidal volume): edema and hemorrhage. Right (adalimumab + high tidal volume): minimal congested areas. Vt - tidal volume; ADA - adalimumab; AST - alveolar septal tickening

After 3 hours of MV, gross inspection showed that all lungs from the LVt group had a normal appearance, whereas the lungs from the HVt group had hemorrhage, congestion, and edema (Figure 1C).

**Figure 2:**
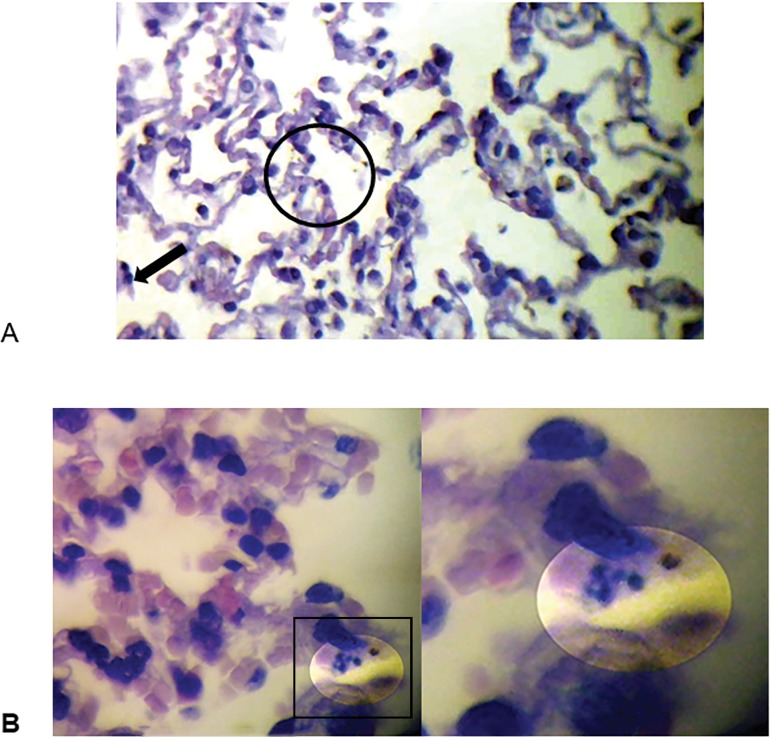
Microscopic lung images (A) Detached alveolar coat cell (arrow) and fibrous intraalveolar walls in the process of repair (circle). Microscopic images from the adalimumab + high tidal volume group. (B) Apoptotic bodies recognized and digested by alveolar macrophages. This phenomenon is triggered by specific cell membrane receptors. Microscopic images from the adalimumab + high tidal volume group.

**Figure 3:**
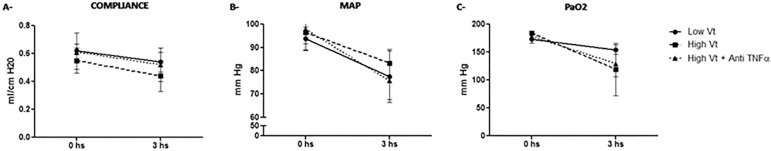
Monitoring during the experimental procedure. Changes in thoracic system compliance, mean arterial pressure and the partial pressure of arterial oxygen: ventilation with a high tidal volume was associated with decreased thoracic system compliance at 3 hours (p = 0.04), and comparison of the high tidal volume group versus the adalimumab + high tidal volume group showed no differences regarding lung compliance (p = 0,26). All groups presented a mean arterial pressure drop over the 3 hours of the study with no significant differences. Ventilation with a high tidal volume was associated with hypoxemia compared with ventilation with a low tidal volume (p = 0.018), while the adalimumab-treated group showed no statistically significant differences at 3 hours compared with the high tidal volume group (p = 0.42). MAP - mean arterial pressure; PaO2 - partial pressure of arterial oxygen; Vt - tidal volume; TNF-α - tumor necrosis factor alpha.

On microscopic inspection, the lungs in the HVt group showed alveolar and interstitial space polymorphonuclear leucocyte infiltration, with the presence of hyaline membranes and intraalveolar proteinaceous debris; detached alveolar coat cells; and fibrous intraalveolar walls during the process of repair (Figures 1B, 1C and 3A). Low percentages of apoptotic bodies were seen in the LVt group and the pretreated HVt group (Figure 2B). The mean histological lung injury score was higher in the HVt group than in the LVt group (0.030 *versus* 0.0051, respectively, p = 0.003). The mean histological score was higher in the HVt group than in the pretreated HVt group (0.030 *versus* 0.0052, respectively, p = 0.003) (Figure 1A). The pretreated HVt group had an improved histological score, mainly due to a significant reduction in leukocyte infiltration. Worsening in thoracic system compliance in the HVt group and pretreated HVt group was seen at 3 hours (p = 0.04) (Figure 3 and Table 1).

**Table 1:** Compliance analysis: lung compliance in the three groups (low tidal volume, high tidal volume, and adalimumab + high tidal volume) at baseline and 3 hours after intervention

	Baseline	3 hours
Low Vt	0.61 ± 0.08	0.53 ± 0.05
High Vt	0.54 ± 0.05	0.44 ± 0.06
High Vt + ADA	0.57 ± 0.08	0.54 ± 0.17

Vt - tidal volume; ADA - adalimumab. Compliance is expressed as the average ± standard deviation (N = 6 per group).

The W/D ratio in the HVt group was higher than that in the LVt group (p = 0.0001); the W/D ratio differed between the pretreated HVt group and HVt group (p = 0.0001) (Table 2).

**Table 2:** Lung edema evaluation

	1	2	3	4	5	6
Low Vt	0.05	0.067	0.033	0041	0.052	0.037
High Vt	4.40	4.91	3.92	4.20	4.78	4.41
High Vt + ADA	1.51	1.83	1.28	1.42	1.60	1.52
			**3 hours**			
		Low Vt	0.046 ± 0.012			
		High Vt	4.43 ± 0.36			
		High Vt + ADA	1.51 ± 0.18			

The ratio of wet weight to dry weight in the high tidal volume group was higher than that in the low tidal volume group; the ratio of wet weight to dry weight differed between the adalimumab + high tidal volume group and the high tidal volume group. Vt - tidal volume; ADA - adalimumab. The wet weight-to-dry weight ratio is expressed as the average ± standard deviation (N = 6 per group).

No statistically significant differences in hemodynamic parameters (MAP) or the PaO_2_ were observed between the pretreated HVt group and the HVt group (p = 0.42) (Figure 3). The partial pressure of arterial carbon*dioxide* did not differ between these two groups. At the end of the protocol, oxygenation tended to deteriorate in the HVt and pretreated HVt groups; the partial pressure of oxygen (PO_2_) remained significantly higher in the LVt group than in the HVt group (p = 0.018) and the pretreated HVt group (p = 0.007) (Figure 3).

## DISCUSSION

In this experimental study, the duration of MV was much shorter than that required by patients with lung injury; however, it is comparable with the MV time used in most published experimental VILI studies. We used two ventilation strategies: the LVt group was ventilated with a frequently used protective approach (Vt of 8mL/kg and PEEP of 5cmH_2_O), which is generally considered harmless,^(22)^ while the HVt and pretreated HVt groups were ventilated with a Vt of 35mL/kg and no PEEP, which is considered injurious. At randomization, the PaO_2_ was normal in all groups, suggesting that there was little or no lung injury at baseline. After 3 hours of MV, lung samples from the HVt group had diffuse alveolar damage, whereas those from the LVt group did not. In contrast, proportional changes in the PaO_2_/FiO_2_ and lung compliance were minor. These changes did not result in increased compliance as expected, probably due to the short period of MV performed in this study, which may not reflect the expected pulmonary mechanical changes. Compared to the HVt group, the pretreated HVt group showed no significant differences with respect to oxygenation and pulmonary mechanics; however, histological findings driven by MV were minimized in the pretreated HVt group. Compared to the HVt group, the pretreated HVt group had lower histological lung injury due to decreased leukocyte infiltration into the alveolar space and pulmonary interstitium and a significant reduction hyaline membrane production, with similar impacts on septal thickening and proteinaceous material.

Adalimumab completely prevented structural lung injury and significantly decreased the histological lung injury score compared to HVt alone. Little apoptosis was observed in the adalimumab + HVt group (Figure 2A). Cell death occurs in two ways: necrosis and apoptosis. Necrosis can be observed when tissues become ischemic and the PaO_2_ falls drastically; this mechanism is probably less common in the lungs than in other tissues because of the double pulmonary circulation.^(16,23-26)^

In general, apoptosis occurs without the release of cellular products into the intercellular space, whereas necrosis is associated with cellular inflammation, ruptured membranes, and the release of intracellular products into the local environment. This process is extremely fast, so the number of apoptotic leukocytes visible in a swollen tissue is generally low and probably underestimates the actual degree of apoptosis^(27)^ (Figure 2B). The destruction of the alveolar walls, the activation of fibroblasts, and the production of collagen lead to fibrosis during the repair process (Figure 2A).

We quantified the number of apoptotic polymorphonuclear leukocytes through their characteristic pattern of nuclear condensation after hematoxylin and eosin staining. The finding of apoptosis in the HVt group supports the argument that stimulation with TNF-α triggers a proinflammatory response as a proapoptotic signal.^(28-30)^ In a mouse model, Bertok et al.^(14)^ found substantial improvement in the PaO_2_/FiO_2_ and permeability of the respiratory and alveolar barriers when an anti-TNF-α agent was administered intratracheally at the beginning of MV. In our study, we administered adalimumab intraperitoneally 24 hours before the beginning of MV. We decided to use this strategy because the absorption and distribution of adalimumab after subcutaneous administration of a single dose of 40mg in humans is slow, reaching peak plasma concentrations 5 days after administration; we expected the intraperitoneal route to be faster, especially given the high metabolism of rats.

Moreover, intraperitoneally administered adalimumab has been shown to minimize the inflammatory response in an experimental model of pancreatitis in rats, and we chose our dose based on the results of that study.^(20)^

Tumor necrosis factor alpha is particularly important in lung injury because the lungs contain a large reservoir of cells producing TNF-α.^(16)^ Our experiment was performed in healthy rats; the benefits of adalimumab may be even greater in humans who require prolonged MV treatment. Infliximab and adalimumab, although sharing the same mechanism of action, are different molecules, and infliximab has been studied in rats and shown to minimize pulmonary fibrosis due to bleomycin. In our study, lung injury associated with volutrauma was minimized in rats who underwent MV.^(28)^

In the present study, PaO_2_/FiO_2_ levels were similar in the LVt group and the HVt group, probably because the period of MV was too short to affect oxygenation. A longer experimental period may show a difference between these two groups and a difference between the HVt group and the pretreated HVt group.

Retrospectively, as limitations of this study, we could have measured the levels of other inflammatory mediators that could be affected by inhibiting TNF-α. Another limitation related to apoptosis analysis is the lack of quantitative measurement methods (e.g., TUNEL staining) that may have better characterized apoptosis in this study.

A clinical trial with intravenous anti-TNF-α antibody administration in patients with septic shock found no clinical benefit,^(31)^ but a recent meta-analysis suggests that immunotherapy with a monoclonal anti-TNF-α antibody in patients with sepsis reduces overall mortality and anti-TNF-α therapy may improve survival in patients with shock.^(32)^ The association between lung injury and sepsis represents an opportunity for the use of these drugs.

Studying the hypothesis that patients previously treated with adalimumab or similar immunomodulators have a reduced risk of VILI may create a window of opportunity for other clinical applications, and these findings may contribute to the understanding of the pathophysiology of lung injury. Additionally, these findings may help in situations where there is a high risk of lung injury during elective surgeries, such as lung transplant or prolonged MV supported surgeries.

## CONCLUSION

Intraperitoneal administration of adalimumab 24 hours before injurious mechanical ventilation significantly reduced ventilator-induced lung injury in this experimental rat model. This reduction was associated with hemodynamic improvement but had no impact on oxygenation or lung compliance. Adalimumab prevented the recruitment of polymorphonuclear leukocytes into the alveoli and pulmonary interstitium. Inhibition of tumor necrosis factor alpha may be a promising adjunctive approach to protective ventilation in an attempt to avoid ventilator-induced lung injury.
